# SNA-modified antisense oligonucleotides: A new pathway for renal targeting?

**DOI:** 10.1016/j.omtn.2025.102476

**Published:** 2025-02-17

**Authors:** Bernhard Dumoulin, Ken Yamada, Katalin Susztak

**Affiliations:** 1 Department of Medicine, Renal Electrolyte and Hypertension Division, University of Pennsylvania, Philadelphia, PA, USA; 2 Institute of Diabetes Obesity and Metabolism, University of Pennsylvania, Philadelphia, PA, USA; 3 Department of Genetics, University of Pennsylvania, Philadelphia, PA, USA; 4 Penn-CHOP Kidney Innovation Center, University of Pennsylvania, Philadelphia, PA, USA; 5 RNA Therapeutics Institute, UMass Chan Medical School, Worcester, MA 01655 USA

## Abstract

Antisense oligonucleotides (ASOs) have emerged as a powerful class of therapeutics capable of suppressing gene expression with remarkable specificity. However, the clinical applications of ASOs have been limited by delivery challenges and toxicities, particularly when repeated or high dosing is required. In the study by Tsuboi et al., the authors present serinol nucleic acid (SNA)-modified gapmer ASOs that target the sodium-glucose cotransporter 2 (SGLT2) in mouse kidneys. With promising results, these SNA-modified ASOs display dose-dependent efficacy, prolonged knockdown, and importantly, a more favorable safety profile in both the kidney and liver compared with the 2′-MOE-modified counterpart. While some caveats remain—particularly around high-dose toxicity—these findings open the door to an approach that couples potency with improved tolerability, thereby highlighting SNA-modified ASOs as a potential next-generation platform for renal diseases.

## Main text

Nucleic acid therapeutics, including antisense oligonucleotides (ASOs) and small interfering RNAs (siRNAs), have revolutionized the way we approach diseases driven by aberrant gene expression. One of the earliest successes in the ASO field was the drug nusinersen, which targets the *SMN2* gene in spinal muscular atrophy. Meanwhile, other ASOs have been approved or are under investigation for conditions ranging from familial hypercholesterolemia to transthyretin-mediated amyloidosis. Despite these gains, the kidney—an organ central to filtration, fluid balance, and electrolyte homeostasis—has not seen equivalent strides in ASO-based therapies. This absence is notable considering that systemically administered oligonucleotides often show significant uptake in kidney proximal tubular cells, suggesting a theoretical advantage for treating renal conditions.

The biggest hurdles to realizing the full therapeutic potential of ASOs in the kidney relate to safety and delivery. Chemical modifications such as phosphorothioate backbones, 2′-*O*-methoxyethyl (2′-MOE), and locked nucleic acid (LNA) have each aimed to bolster the pharmacological properties of oligonucleotides—namely, their metabolic stability, cellular uptake, and target-binding affinity. However, these modifications can carry drawbacks, including off-target effects and organ toxicity. Hepatotoxicity and, in some cases, nephrotoxicity have been described for certain modifications, dampening enthusiasm and complicating regulatory paths. Thus, the need for innovative chemistries that improve stability and delivery without incurring severe toxicities remains acute.

Serinol nucleic acid (SNA) has been introduced as one such novel approach. Asanuma’s group has extensively studied the chemical and biophysical properties of “acyclic” artificial nucleic acid including SNA and reported the impact of SNA modification on anti-miR oligonucleotide, splice-switching oligonucleotide, and siRNA.^1^^,^^2^^,^^3^^,^^4^ Prior *in vitro* studies showed that SNA-modified nucleic acids exhibit enhanced nuclease resistance and maintain strong binding to complementary RNA.^3^^,^^4^ However, *in vivo* data demonstrating the efficacy of SNA in the context of RNAseH-dependent and safety have been lacking. Tsuboi et al. address this gap by administering SNA-modified gapmer ASOs subcutaneously in mice to knock down sodium-glucose cotransporter 2 (SGLT2), a proximal tubular protein that reabsorbs filtered glucose. Because SGLT2 inhibitors have become mainstays in the treatment of diabetic kidney disease and heart failure, a selective knockdown strategy could serve as both a proof of concept and a way to further explore the therapeutic potential of gene silencing for kidney targets.

One of the central discoveries in this study is that a single SNA modification at each end of the ASO, designated as SNA2-ASO, effectively reduces *Sglt2* expression in the mouse kidney, thereby inducing substantial urinary glucose excretion.^5^ The investigators report that moderate doses of SNA2-ASO, ranging from 1 to 10 mg/kg/day, produce results that are either comparable to or surpass those of standard DNA-ASO controls and nearly match the potency observed with 2′-MOE-modified gapmer ASOs.^5^ This capability to achieve robust gene knockdown across this dose range suggests that SNA2-ASO can deliver potent effects while possibly minimizing adverse events, offering a promising strategy for kidney-targeted therapy.^5^

In addition to demonstrating strong target knockdown, SNA2-ASO exhibits a more favorable safety profile than 2′-MOE-modified ASOs, which have been associated with dose-limiting hepatotoxicity in certain contexts. In the present study, repeated administration of 2′-MOE-ASOs led to significantly elevated liver enzymes and mild tissue changes such as inflammation or fibrosis, whereas mice treated with SNA2-ASO showed only mild or negligible elevations in markers of liver injury.^5^ These distinctions in toxicity also manifested in the kidney, where 2′-MOE-ASOs elicited greater increases in injury biomarkers, including urinary neutrophil gelatinase-associated lipocalin and the proximal tubule stress marker KIM-1, despite demonstrating less renal uptake than SNA-modified ASOs. These findings underscore that both the chemical structure and the distribution patterns of ASOs determine the extent of organ-specific side effects, and they highlight the therapeutic advantage conferred by adopting SNA-based modifications.

Just as important as tolerability is the durability of gene knockdown, and this study indicates that SNA2-ASO maintains a longer-lasting suppression of its target than standard DNA-ASOs. By day 18 following administration, the SNA-modified cohorts (SNA2, and SNA4 consisting of two SNA modifications at both ends) continued to display marked reductions in *Sglt2* expression, whereas their unmodified counterparts largely lost efficacy.^5^ This extended duration of action has the potential to improve patient adherence and overall treatment safety if similar results can be replicated in clinical settings. Fluorescence imaging provided additional mechanistic insight, revealing that SNA-modified ASOs rapidly accumulate in the renal cortex, specifically in proximal tubular cells, whereas 2′-MOE-ASOs exhibited more pronounced accumulation in the liver, which may account for their higher observed toxicity. For applications that require a kidney-focused approach, the ability to direct a larger fraction of ASOs to proximal tubules is especially beneficial, and these collective findings suggest that SNA-modified oligonucleotides could serve as a potent and safer alternative for future renal therapeutics.

These results align with the broader goal of “organ-selective” nucleic acid therapy, where the chemical modifications improve trafficking to specific tissues while limiting accumulation in off-target sites. For ASO-based interventions in kidney diseases^6^—be they diabetic nephropathy, autosomal dominant polycystic kidney disease, or glomerulonephritides—being able to efficiently deliver and retain the drug in proximal tubules or other relevant kidney compartments could enhance both efficacy and safety.

Moreover, Tsuboi et al. highlight a nuance that not all SNA configurations are equally safe. While the SNA4 construct achieved robust knockdown, its toxicity profile was marginally more concerning than that of SNA2. Such dose-limiting adverse events underscore that even subtle changes in ASO chemistry can greatly influence toxicity. Thus, “less may be more” in the case of SNA modifications, with SNA2-ASO appearing to strike a more balanced efficacy-toxicity ratio.

High-dose toxicity remains a significant concern, even though SNA2-ASO showed a better safety profile compared to 2′-MOE-ASO at moderate dosing. At very high doses of 30 mg/kg/day, there were clear indications of inflammation and liver damage, underscoring the need for rigorous evaluation of the therapeutic window in larger animal models, and eventually, in human trials. Establishing the maximum tolerated dose and refining the dose-response relationship will be central to ensuring that SNA2-ASO and similar constructs can be deployed clinically with acceptable risk-benefit ratios.

Another pressing question involves sequence-specific off-target effects, which are a potential pitfall for any gapmer ASO strategy. Because gapmer ASOs work through RNase H1-mediated mRNA cleavage, ensuring that the intended target is the only RNA significantly reduced is crucial for both efficacy and safety. Going forward, investigators will need to conduct comprehensive transcriptome-wide off-target analyses and toxicogenomic studies to validate the precision of SNA2-ASO and to rule out deleterious effects that might arise from partial complementarity with non-target transcripts.

A further area of inquiry concerns the generalizability of these findings to therapeutic targets beyond SGLT2. Although SGLT2 is an excellent proof of concept due to the ease of measuring its functional decline through glucosuria, other renal targets, particularly those implicated in inflammation, fibrosis, or genetic kidney disorders, stand to benefit from a similar approach. Demonstrating efficacy against a broader spectrum of renal disease pathways could cement the role of SNA2-ASO in the nucleic acid therapeutics arsenal and enhance its clinical utility.

Finally, the field will be watching for comparative data on how SNA fares against other emerging modifications. SNA represents one member of an expanding set of nucleic acid chemistries that also includes LNA, 2′-MOE, bicyclic nucleic acid, phosphoryl guanidine diester, mesyl phosphoramidate, and amide backbones. Head-to-head comparisons within relevant disease models would illuminate relative differences in potency, specificity, safety, and cost. Such studies would not only clarify where each modification excels but also offer clearer guidelines on how best to design nucleic acid therapeutics for a range of indications, including those centered on the kidney.

Looking forward, if additional preclinical studies confirm a favorable safety profile, then SNA-modified ASOs could advance rapidly, particularly for kidney indications with few therapeutic options. The capacity to deliver potent ASOs without extensive hepatic or renal toxicity may also open possibilities for combination strategies where an SNA-ASO is used alongside other agents, such as SGLT2 inhibitors, renin-angiotensin-aldosterone system blockers, or novel biologics.

Ultimately, what is most appealing here is the prospect of “kidney-centric” precision therapy. Renal diseases with strong genetic underpinnings, or those that rely on upregulated transcripts in proximal tubules, could benefit substantially from an approach that localizes an ASO to the site of pathology. If SNA2-ASOs indeed prove safer across multiple preclinical species and eventually in human trials, then they may help transcend some of the biggest hurdles in the field of nucleic acid therapeutics.

### Conclusion

In summary, Tsuboi et al. offer compelling evidence that SNA-modified ASOs can achieve robust, durable gene knockdown in the kidney while mitigating some of the toxicity issues plaguing other chemical modifications. Their demonstration of effective SGLT2 suppression, alongside improved liver and kidney safety profiles, underscores the promise of SNA as a versatile backbone for the next generation of renal-focused therapeutics. As the field moves from proof-of-principle studies toward real-world clinical applications, SNA2-ASOs hold the potential to transform how we treat a wide spectrum of kidney diseases and possibly beyond.

## Acknowledgments

Work in the Susztak lab was supported by the National Institutes of Health grant nos. P50DK114786, DK076077, DK087635, DK132630, and DK105821. B.D. is supported by the Deutsche Forschungsgemeinschaft (DFG; German Research Foundation, grant DU 2449/1-1).

## Declaration of interests

The authors declare no competing interests.
